# Context-dependent oxytocin receptor signaling: from molecular architecture to transcript-level receptor engineering

**DOI:** 10.3389/fnins.2026.1938379

**Published:** 2026-10-06

**Authors:** Benjamin Jurek, Julia J. Winter, Daniel S. Quintana

**Affiliations:** 1 Department of Neurobiology, Ludwig Maximilians-University Munich, Munich, Germany; 2 Neurobiology of Stress Resilience, Max Planck Institute for Psychiatry, Munich, Germany; 3 Systems Pharmacology and Translational Therapeutics, Perelman School of Medicine, University of Pennsylvania, Philadelphia, PA, United States; 4 Epigenetics Institute at the Perelman School of Medicine at the University of Pennsylvania, Philadelphia, PA, United States; 5 Department of Psychology, University of Oslo, Oslo, Norway; 6 K.G. Jebsen Centre for Neurodevelopmental Disorders, University of Oslo, Oslo, Norway; 7 Department of Rare Diseases, Oslo University Hospital, Oslo, Norway

**Keywords:** exon-skipping, gapmer antisense oligonucleotides, G protein coupled receptor (GPCR), intracellular signaling cascades, OXTR, SNP, soluble GPCR, splice variant

## Abstract

The oxytocin receptor (OXTR) is a G protein-coupled receptor that integrates neuroendocrine release, cellular signaling, and behavioral effects across species. Despite extensive research, the mechanistic basis underlying variability in oxytocin's effects remains incompletely understood. This review synthesizes recent advances in OXTR biology and focuses on three biological sources of signaling heterogeneity: variation in receptor sequence and structure, epigenetic and cell-state-dependent regulation, and temporal patterns of receptor activation. It examines targeted manipulation of receptor transcripts as an experimental strategy for testing intracellular mechanisms with behavioral outcomes. Mechanistic studies show that subtle structural changes, including single nucleotide polymorphisms, can bias intracellular signaling toward distinct pathways. Chronic receptor activation induces transcriptional and post-transcriptional reprogramming that can invert behavioral outcomes relative to acute stimulation. Intracellular signaling converges on core pathways, including Ca^2+^-dependent cascades, MAPK signaling, and transcriptional regulators such as CREB and MEF2A, which together shape gene expression, protein synthesis, and cellular morphology. Human studies reveal substantial variability in oxytocin effects, consistent with context-dependent signaling and limited control over intracellular pathways. These observations illustrate the challenge of linking receptor-level processes to behavioral phenotypes and underscore the importance of mechanistic approaches to OXTR signaling. Within this framework, GapmeR-mediated manipulation of OXTR transcripts and CRISPR-based genetic or epigenetic approaches are discussed as preclinical research strategies for testing receptor-level mechanisms. These strategies complement classical biased-ligand-pharmacology and support a model in which OXTR functions as a context-sensitive signaling hub, determined by receptor structure, activation history, and intracellular state.

## Introduction

Oxytocin (OXT) has long been regarded as a central regulator of social behavior, emotional processing, and stress responsivity. Early studies established its roles in parturition and lactation, while subsequent work extended its functional repertoire to include social bonding, anxiety modulation, and cognitive processes (Jurek and Neumann, 2018). However, the classical view of OXT as a uniformly prosocial and anxiolytic neuropeptide has been progressively challenged. Accumulating evidence demonstrates that OXT can produce divergent and, in some cases, opposing behavioral outcomes depending on context, individual variability, and experimental conditions (Bartz et al., 2011; Jurek and Meyer, 2020).

At the molecular level, these effects are mediated by the OXT receptor (OXTR), a class A G protein-coupled receptor capable of engaging multiple intracellular signaling cascades. The OXTR exhibits substantial signaling flexibility, coupling to Gq/11-, Gi/o-, and β-arrestin-dependent pathways, thereby enabling a broad range of downstream responses (Busnelli and Chini, 2018; Gimpl and Fahrenholz, 2001; Jurek and Neumann, 2018). This flexibility is further modulated by receptor-intrinsic factors such as genetic variation and structural modifications, as well as by extrinsic factors including ligand concentration, temporal dynamics of receptor activation, and cell-type specificity.

In recent years, research on the significance of epigenetic modifications to the *OXTR* gene has been rapidly evolving (Nance et al., 2025). Variability in *OXTR* methylation has been linked to various psychiatric disorders and neurodevelopmental conditions including autism (Gregory et al., 2009; Puglia et al., 2015). Further, differences in therapeutic effects of synthetic OXT could be explained by epigenetic mechanisms of *OXTR* regulation (Nance et al., 2025).

Parallel to mechanistic advances, human OXT research has produced a complex and often inconsistent body of findings, where replication remains variable and effect sizes are often modest (Quintana et al., 2021). These discrepancies underscore a central challenge in the field: the lack of a coherent mechanistic framework linking receptor-level signaling to behavioral outcomes.

This review integrates findings across four interrelated domains: receptor structure, cell-type-specific signaling, temporal signaling dynamics, and targeted experimental manipulation. The first three describe biological sources of heterogeneity. GapmeR- and CRISPR-based approaches are considered separately as mechanistic research tools that can test causality in cellular and preclinical *in vivo* systems, rather than as explanations for naturally occurring human variability. The specific contribution of this review is to integrate receptor architecture, cell-type-specific signaling, and receptor binding kinetics with a methodological framework (i.e., transcript-level engineering) so that OXTR system output will become predictable in its full variability.

## Receptor architecture and signaling flexibility

The functional versatility of the OXTR is rooted in its molecular architecture. As a seven-transmembrane GPCR, the receptor transduces extracellular ligand binding into intracellular signaling through conformational changes that enable interaction with G proteins and scaffolding molecules. Canonical signaling involves Gq/11-mediated activation of phospholipase C, leading to inositol trisphosphate production and intracellular calcium (Ca^2+^) release (Busnelli and Chini, 2018; Devost et al., 2008; Jurek and Neumann, 2018).

However, OXTR signaling extends beyond this classical pathway. The receptor can couple to multiple G protein subtypes and recruit β-arrestin, enabling G protein-independent signaling. Structural studies have demonstrated that ligand binding stabilizes distinct receptor conformations, thereby determining downstream signaling bias (Meyerowitz et al., 2022; Waltenspühl et al., 2022). In the inactive human OXTR–retosiban structure, antagonist recognition is organized within the transmembrane bundle, and an extrahelical cholesterol molecule occupies a site between transmembrane helices IV and V, illustrating a direct receptor–lipid contact (Waltenspühl et al., 2020). Active-state cryo-EM structures of OXT-bound human OXTR show that activation involves extracellular ligand-capping interactions, Mg2+-coordinated ligand–receptor contacts, disruption of transmembrane helix 7, and outward movement of transmembrane helix 6 to permit G-protein engagement (Meyerowitz et al., 2022; Waltenspühl et al., 2022). Together, these studies identify the ligand-binding pocket, extracellular loops, cation-coordination site, transmembrane activation microswitches, intracellular effector interface, and surrounding lipid environment as distinct structural levels at which OXTR output may be modulated.

Neuronal OXTR phosphorylation, internalization, GRK2/3/6-dependent desensitization and recycling further modulate signaling dynamics (Conti et al., 2009; George et al., 2024; Reversi et al., 2005; Rimoldi et al., 2003). Quaternary receptor organization represents an additional layer of regulation. Human OXTR, vasopressin V1a, V2, dopamine receptor D2, as well as serotonin receptors 2A and 2C can form homo- and hetero-dimers in various expression systems (Borroto-Escuela et al., 2022; Terrillon et al., 2003). However, most evidence derives from overexpression, co-immunoprecipitation, or resonance-energy-transfer assays, and the consequences for pathway selection in native human brain cells remain unresolved. We therefore consider dimerization a plausible context-dependent modifier rather than an established cause of behavioral variability. These processes regulate both the duration and intensity of signaling, enabling the receptor to encode temporal information into intracellular responses. Together, these features establish the OXTR as a highly dynamic signaling platform rather than a simple on–off switch.

## Genetic and epigenetic variation and signaling bias

Genetic variation provides one route through which receptor structure can alter signaling. In monoclonal HEK293 cell lines stably expressing either wild-type human OXTR or the non-synonymous A218T (rs4686302) variant, A218T increased receptor stability, altered Ca^2+^ dynamics, and attenuated ERK1/2–MAPK activation (Meyer et al., 2022). These data demonstrate, under a controlled heterologous-expression context, that a coding substitution can modify receptor-proximal signaling kinetics and pathway balance.

These effects extend beyond second-messenger signaling. Transcriptomic profiling of A218T-expressing cells revealed broad gene expression changes, including enrichment of Ca^2+^ signaling, MAPK cascades, cellular morphology, and mitochondrial pathways (Meyer et al., 2022), indicating multi-level remodeling of signaling networks. However, not all *OXTR* polymorphisms appear to act through direct transcriptional effects on the OXTR mRNA. In a recent functional study, only rs1042778 significantly altered reporter expression, whereas rs4686302, rs2254298, and rs237887 showed no measurable change of OXTR mRNA (Ma et al., 2025), suggesting alternative regulatory mechanisms.

Consistent with this, regulatory variation at the *OXTR* locus appears to be distributed across a broader cis-regulatory landscape. Enhancer-mapping studies have identified multiple *OXTR*-associated regulatory elements, indicating that receptor expression is governed by complex, cell type-specific regulatory architecture rather than single polymorphisms (Laboy Cintrón et al., 2025). Accordingly, many trait-associated SNP effects may reflect linkage to regulatory regions rather than direct coding changes.

Human basal-forebrain tissue data further support this interpretation. Although OXTR mRNA and receptor binding were altered in specific regions in autism, commonly studied OXTR SNPs, including rs4686302, did not predict these regional changes (Dayley et al., 2026), indicating that receptor mRNA expression and availability are not straightforwardly determined by individual variants. Interestingly, variability in DNA methylation of the *OXTR* gene has been shown to influence transcription levels and has been linked to psychiatric disorders in humans (Nance et al., 2025; Puglia et al., 2015). This indicates that cell composition, cis-regulatory context, and epigenetic state can dominate receptor abundance *in vivo*.

Similarly, in clinical populations with schizophrenia, *OXTR* polymorphisms have been linked to symptom severity and treatment response (Lv et al., 2024), but the overall evidence suggests a more complex model. Rare coding variants can directly bias signaling, whereas common SNPs likely exert context-dependent effects through regulatory architecture and cellular environment. This framework helps reconcile strong genetic associations with the modest and variable effects observed in human studies.

## Temporal dynamics: acute vs. chronic receptor activation

In addition to receptor structure, the temporal pattern of OXTR activation is a critical determinant of signaling outcome. Acute OXT exposure within the paraventricular nucleus (PVN) is consistently associated with anxiolytic effects and is mediated by rapid intracellular signaling mechanisms, including Ca^2+^ mobilization, MEK1/2–ERK1/2 activation, and eEF2-dependent protein synthesis (Jurek et al., 2012; Martinetz et al., 2019). These processes primarily operate on short timescales and modulate neuronal activity and local circuit function without inducing substantial changes in receptor composition or transcript processing. In this context, OXTR signaling is largely confined to classical second messenger pathways and their immediate downstream effectors.

In contrast, prolonged OXTR activation engages a distinct signaling regime that extends beyond transient eEF2 activation. In our study (Winter et al., 2023), continuous intracerebroventricular OXT administration over 14 days resulted in increased anxiety-like behavior in rats, in contrast to the anxiolytic effects observed following acute administration. This behavioral effect was reversible after cessation of treatment, indicating that chronic OXTR activation induces persistent but dynamic molecular adaptations rather than irreversible changes. The anxiogenic phenotype was accompanied by activation of a specific intraneuronal or glial signaling cascade in the PVN, characterized by increased phosphorylation of MEK1/2 and ERK1/2, as well as enhanced expression and activity of the transcription factor MEF2A. Notably, although also starting with MAPK recruitment, acute OXT administration did not recruit MEF2A signaling, suggesting that its activation reflects a temporally dependent transition in OXTR-MAPK signaling rather than a general downstream consequence of receptor stimulation.

A key finding of our study is that chronic OXTR activation alters transcript processing of downstream receptor systems. Specifically, prolonged OXT exposure shifted the splicing pattern of corticotropin-releasing factor receptor 2α (*Crfr2*α) from the membrane-bound isoform (*mCRFR2*α) toward the soluble splice variant (*sCRFR2*α). This shift was observed at both mRNA and protein levels in the PVN and was not detected following acute OXT treatment. These results indicate that alternative splicing represents a central mechanism through which sustained OXTR signaling modifies downstream receptor function (Winter et al., 2023).

Mechanistically, this splicing event is linked to MEF2A activation. Chromatin immunoprecipitation and knockdown experiments demonstrated that MEF2A binds to the *Crfr2*α gene and contributes both to its transcription and to splice-site selection. In particular, MEF2A association with exon 6 appears to influence alternative splicing, thereby coupling transcriptional regulation with RNA processing. These findings are consistent with previous evidence that transcription factors can coordinate transcription and co-transcriptional splicing through interactions with chromatin-associated regulatory complexes. In this framework, chronic OXTR signaling engages an ERK1/2–MEF2A pathway that extends beyond transcriptional control to regulate receptor isoform composition (Winter et al., 2023).

The functional relevance of this splicing shift is supported by the distinct properties of the *Crfr2*α isoforms. The membrane-bound receptor is associated with anxiolytic signaling, whereas the soluble variant lacks the transmembrane domain and is therefore not inserted into the plasma membrane. However, sCRFR2α retains ligand-binding capacity for CRF-related peptides, including CRF and urocortins. This raises the possibility that sCRFR2α acts as a ligand-binding protein in the extracellular space, thereby reducing ligand availability for membrane-bound CRFR2α and attenuating its signaling. Within this framework, chronic OXTR activation modifies stress-related signaling not by directly altering CRF production, but by reshaping receptor availability and ligand distribution within the local microenvironment (Winter et al., 2023).

Consistent with this interpretation, sCRFR2α was detected in cerebrospinal fluid, and its levels positively correlated with anxiety-like behavior. *In vitro* experiments further demonstrated that chronic OXT exposure reduces membrane-associated CRFR2α while increasing the release of the soluble isoform from hypothalamic neurons. These findings suggest that alternative splicing of *Crfr2*α not only alters receptor composition at the cellular level but also generates a diffusible component that may influence signaling beyond the immediate site of production (Winter et al., 2023).

Acute and chronic OXTR activation therefore share ERK1/2 as a proximal signaling node but diverge at the level of downstream effectors. Acute activation engages eEF2-dependent protein synthesis associated with anxiolysis, whereas sustained activation recruits MEF2A, promotes alternative splicing of *Crfr2*α, and shifts signaling toward an anxiogenic state. The acute eEF2-dependent mechanism was demonstrated in male rats, while chronic OXT increased anxiety-like behavior in both sexes, with greater sensitivity in females.

## Integration of signaling pathways

At the mechanistic level, OXTR signaling is most appropriately conceptualized as a multi-layered and highly integrated signaling network rather than a single canonical cascade (Figure 1). The receptor belongs to the class A GPCR family and is capable of coupling to multiple intracellular effectors, including Gq/11-dependent phospholipase C signaling, Gi/o-mediated pathways, and β-arrestin-associated signaling modules. The relative contribution of these pathways is determined by receptor conformation, ligand context, membrane environment, and cell type (Busnelli and Chini, 2018; Devost et al., 2008; Gimpl and Fahrenholz, 2001; Jurek and Neumann, 2018). Structural analyses of the active human OXTR in complex with OXT and G proteins further support the notion that signaling output is governed by ligand-stabilized receptor conformations and specific receptor–effector interfaces, rather than a binary “occupied vs. unoccupied” receptor state (Meyerowitz et al., 2022; Waltenspühl et al., 2022).

**Figure 1:**
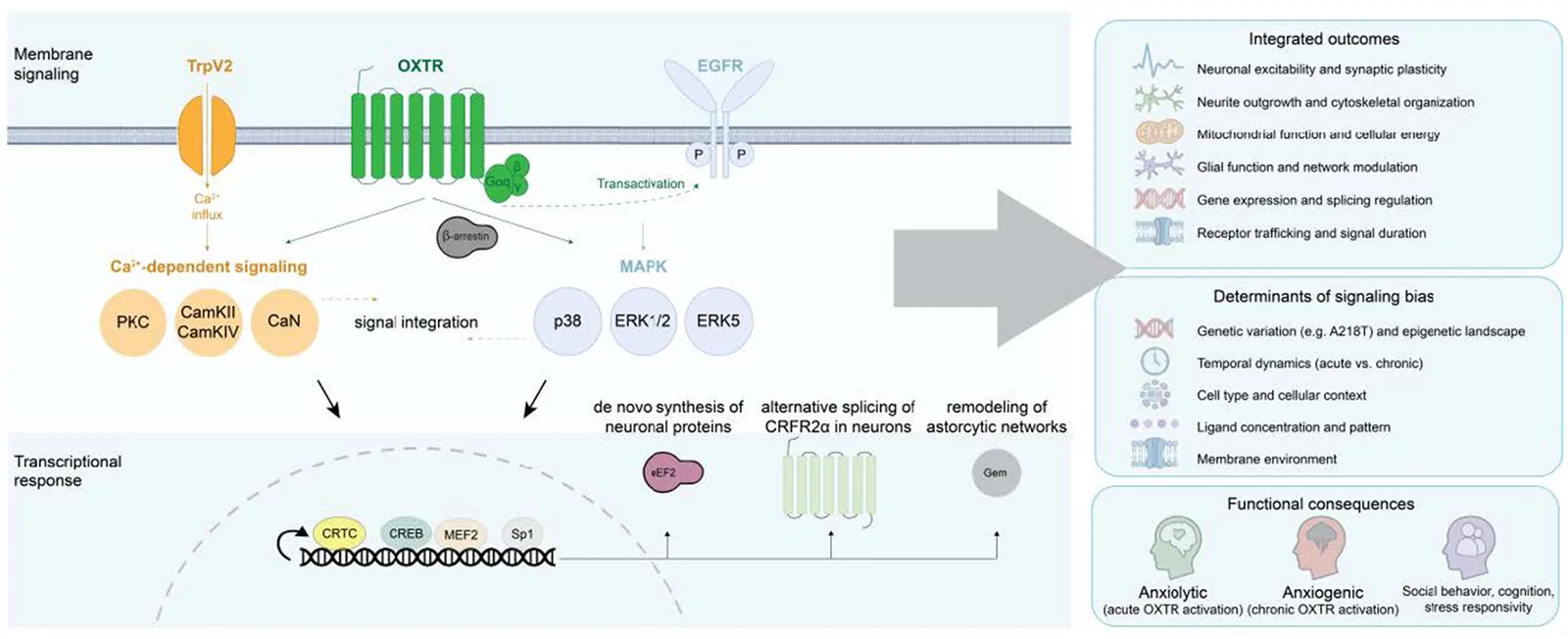
OXTR-mediated signaling cascades: from receptor activation to cellular and functional outcomes. Schematic representation of OXTR activation, which promotes insertion of TrpV2 channels into plasma membrane, triggering Ca2+-dependent signaling through PKC, CaMKII/IV and CaN. OXTR activation also induces EGFR transactivation that leads to activation of MAPK pathways including ERK1/2, ERK5, and p38. These signaling cascades converge on the CREB-CRTC/MEF2 transcriptional complex, resulting in increased expression of target genes. Additionally, in astrocytes OXTR activation leads to activation of a novel Sp1-Gem pathway resulting in remodeling of astrocytic networks. Receptor regulation is further achieved by phosphorylation, β-arrestin recruitment, internalization, and recycling.

Cell identity determines the availability of G proteins, GPCR kinases, arrestins, ion channels, cytoplasmic and nuclear signaling kinases, and transcription factors. For instance, in hypothalamic neuronal cells (H32) and tissue, OXTR activation induces intracellular Ca^2+^ mobilization via PLC/IP3-dependent release from intracellular stores, while also facilitating extracellular Ca^2+^ influx, including TRPV2-mediated mechanisms (Jurek and Neumann, 2018; Meinung et al., 2025; van den Burg et al., 2015). The source, duration, and temporal dynamics of the Ca^2+^ signal are critical determinants of downstream pathway engagement. Experimental evidence demonstrates that structural alterations of the receptor, such as the A218T variant, modify Ca^2+^ signaling kinetics and amplitude, thereby shifting the balance between downstream signaling pathways (Meyer et al., 2022). In the same context, altered Ca^2+^ dynamics were associated with differential expression of Ca^2+^ channel-related genes, including *TRPC6, TRPC3*, and *CACNA1C*, directly linking receptor-level biophysical properties to transcriptomic remodeling (Meyer et al., 2022), whereas in rat skeletal muscle, a non-canonical OXTR-Ca^2+^-Akt pathway contributes to anti-proteolytic effects (Santos et al., 2023).

Downstream of Ca^2+^ signaling, the MAPK network represents one of the most robust and consistently validated signaling axes in OXTR biology. In the hypothalamic paraventricular nucleus, OXT-dependent anxiolysis requires MEK1/2 activation, positioning ERK signaling as a critical mediator of behaviorally relevant output (Jurek et al., 2012). In hypothalamic neuronal cell culture (Jurek et al., 2012, 2015), HEK293 cells (Meyer et al., 2022) and other cell lines (Jurek et al., 2022), ERK1/2 phosphorylation serves as a reliable readout of OXTR activation; however, this response is not invariant. The A218T receptor variant exhibits attenuated ERK1/2 activation despite increased receptor stability and altered Ca^2+^ kinetics, indicating that receptor stabilization does not necessarily enhance canonical signaling strength but can instead bias pathway activation (Meyer et al., 2022).

Importantly, OXTR signaling extends beyond ERK1/2. In myometrial cells, ERK5/MAPK7 has been identified as a downstream effector of OXT signaling, expanding the MAPK repertoire and reinforcing the concept that OXTR signaling is branch-selective and tissue-dependent (Devost et al., 2022). Collectively, these findings emphasize that “MAPK signaling” should not be treated as a single uniform endpoint. Rather, the specific MAPK branch engaged and its temporal dynamics must be considered to accurately interpret signaling outcomes (Devost et al., 2022; Jurek and Neumann, 2018).

These kinase cascades converge on transcriptional regulators that constitute the primary interface between transient receptor activation and long-term changes in cellular state. One well-characterized OXTR-responsive mechanism is the CREB/CRTC3–CRF axis. In the hypothalamic PVN of male rats, OXTR activation delays stress-induced corticotropin-releasing factor (*Crf)* transcription by sequestering the CREB co-factor CRTC3 in the cytosol and reducing its recruitment to the *Crf* promoter, thereby functionally uncoupling stress-induced CREB activation from full coactivator engagement (Jurek et al., 2015). This mechanism provides a clear example of how OXTR signaling can selectively modulate defined transcriptional programs with direct relevance for HPA-axis regulation.

A second major transcriptional pathway involves MEF2A. In hypothalamic neurons, OXT-induced MEF2A activation is mediated by Ca^2+^/calcineurin signaling and is causally linked to neurite remodeling and mitochondrial function (Jurek and Meyer, 2020; Meyer et al., 2020). The functional significance of MEF2A lies in its regulation of a broad gene network controlling cellular morphology and connectivity. This interpretation is supported by studies from Bakos et al. (2013) demonstrating that OXT modulates the expression of cytoskeleton- and plasticity-related proteins, including nestin, MAP2, β-actin, vimentin, cofilin, and drebrin, and regulates neurite outgrowth or cytoskeletal reorganization depending on differentiation state (Havranek et al., 2024; Reichova et al., 2020; Zatkova et al., 2018b) and cellular context, for example, hippocampal neurons (Reichova et al., 2021), SH-SY5Y neuroblastoma cells (Lestanova et al., 2016; Meyer et al., 2020; Vattem et al., 2026), U87MG glioblastoma cells (Zatkova et al., 2018a), primary hypothalamic neurons and glia cells (Winter et al., 2023), hypothalamic H32 (Meyer et al., 2018) and mHypoE-N11 cells (Meyer et al., 2020).

In astrocytes, OXTR signaling engages an additional transcriptional module involving an Sp1–Gem signaling axis. This pathway links OXT exposure to astroglial cytoskeletal remodeling, synaptic coverage, and gap junction dynamics, further extending the impact of OXTR signaling beyond neurons to include glial contributions to circuit function (Meinung et al., 2025).

OXTR signaling also extends beyond transcriptional regulation into translational control. A central mechanism in this context is the activation of eukaryotic elongation factor 2 (eEF2), which directly links receptor activation to *de novo* protein synthesis. In the PVN, OXT-induced eEF2 activation is PKC-dependent, and inhibition of protein synthesis abolishes the anxiolytic effects of OXT, demonstrating that translation represents a behaviorally important readout (Martinetz et al., 2019). Notably, this OXTR-specific pathway is associated with defined protein outputs, including upregulation of neuropeptide Y receptor 5 (*Npy5r*), which contributes functionally to the observed behavioral effects (Martinetz et al., 2019). In this regard, the eEF2 pathway is conceptually analogous to the CREB/CRTC3–CRF mechanism, as both link OXTR signaling to specific downstream gene products with clear functional relevance.

Additional evidence indicates that OXTR can engage non-canonical kinase pathways, including Ca^2+^-dependent activation of PI3K/Akt signaling in peripheral tissues such as skeletal muscle, where OXT exerts anti-proteolytic effects (Santos et al., 2023), or EGFR transactivation and p38 phosphorylation in hypothalamic neurons (reviewed in Jurek and Neumann, 2018). Although the role of PI3K/Akt signaling in central OXTR function remains less well-defined, these findings further expand the signaling repertoire of the receptor.

Overall, gene ontology analyses consistently identify enrichment of pathways related to Ca^2+^ signaling, MAPK cascades, transcriptional regulation, cellular morphology, neuronal connectivity, and mitochondrial function (Meyer et al., 2022). These transcriptomic signatures align closely with experimentally observed neuronal phenotypes, including MEF2A-dependent morphological remodeling and mitochondrial regulation (Meyer et al., 2020). However, OXT-induced effects in one cell type do not necessarily equate to all cell types, and generalizations across different cell lines and tissues should be considered carefully.

Taken together, these findings suggest that ontology-level analyses are fully consistent with receptor-proximal signaling data. Pathways identified at the biochemical level are recapitulated as enriched functional categories in transcriptomic datasets, supporting a coherent multi-level model of OXTR signaling.

For ontology-based analysis of OXT-responsive gene sets, the Gene Ontology knowledgebase provides structured annotations of biological processes, molecular functions, and cellular components accessible through AmiGO and QuickGO (Binns et al., 2009; Carbon et al., 2009). Functional enrichment and pathway mapping can subsequently be performed using g:Profiler and KEGG, respectively (Kanehisa et al., 2025; Kolberg et al., 2023).

## Implications for human oxytocin research

The human OXT research literature is characterized by considerable heterogeneity in results (Alvares et al., 2017; Audunsdottir et al., 2024). While methodological issues related to intranasal oxytocin delivery have certainly contributed to this heterogeneity (Leng and Ludwig, 2016), theoretical considerations have also played a critical role in the design and interpretation of human OXT research (Winterton et al., 2021). Early work suggested that OXT acted in a unitary fashion, as either a “prosocial” (Uvnäs-Moberg, 1998) or “anxiolytic” (MacDonald and Feifel, 2014) hormone. However, more recent work has substantially refined the conceptual framework of OXT signaling by moving away from unitary models and toward more context-dependent and biologically stratified perspectives (Bartz et al., 2011). These contemporary theories emphasize that OXT's behavioral effects are not fixed but depend on interactions between context, individual traits, and underlying neural circuitry, with OXT modulating salience attribution, socio-emotional processing, and reliance on past experience, rather than exerting uniformly prosocial or anxiolytic effects (Quintana, 2026; Yao and Kendrick, 2025; Zhao et al., 2026). Large-scale analyses and meta-analyses have additionally highlighted the conditional nature of OXT's effects, with many reported findings failing to replicate across studies or populations (Declerck et al., 2020; Quintana et al., 2021; Walum et al., 2016). While these more recent theories have helped explain OXT's heterogenous effects, there has been little discussion of the neurobiological mechanisms underlying the conditional nature of OXT's effects. Here we suggest that the heterogeneous outcomes in human OXT research can be better understood as the systems-level manifestation of a receptor whose biological output is inherently dependent on its signaling properties, which are influenced by an interaction of genetic and contextual factors.

Regarding signaling properties, the duration of OXTR's exposure to OXT seems to play an important role in the variability of response. Recent work on repeated OXT administration indicates that its effects extend beyond acute modulation of regional brain activity to include alterations in large-scale network organization, as demonstrated by changes in functional connectivity following multi-week treatment in older adults (Liu et al., 2024). In addition, evidence indicates that endogenous OXT levels are higher 4 weeks after the cessation of long-term OXT treatment, compared to placebo, which is consistent with a feed-forward mechanism (Alaerts et al., 2021). These findings highlight the importance of distinguishing between acute and chronic OXT exposure in human studies, as sustained OXTR activation may engage longer-term plasticity mechanisms analogous to those observed in our chronic preclinical model (Winter et al., 2023). Consistent with this interpretation, meta-analytic approaches have been employed to reassess canonical findings such as OXT-induced modulation of amygdala activity, reflecting the need to quantitatively integrate a literature characterized by variable effect sizes and experimental designs (Guo et al., 2026). While evidence suggests that intranasally delivered OXT can reach the central nervous system, the extent, timing, and distribution of brain exposure remain incompletely characterized (Lee et al., 2020; Striepens et al., 2013). Furthermore, the optimum dosage has yet to be established (Declerck et al., 2020; Quintana, 2022). Given the sensitivity of OXTR signaling to ligand concentration and temporal dynamics, variability in delivery likely translates into variability in intracellular pathway engagement.

Genetic variation also contributes to variability in receptor-level signaling, as genetic variation in the *OXTR* gene has been shown to influence neural responses to OXT, including amygdala activation and social cognition, reflecting receptor-level differences in signaling capacity (Tost et al., 2010). Moreover, OXTR expression patterns in the typical human brain closely mirror neural networks underlying social cognition, among other types of cognitive processing (Quintana et al., 2019). Gene knockout studies in animal models have provided additional insights into the physiological roles of OXTR (Lee et al., 2008; Takayanagi et al., 2005). For instance, across multiple mouse models, OXTR knockout has been associated with impairments in social recognition, social interaction, and maternal behaviors, supporting the central role of receptor signaling in the regulation of social cognition (Macbeth et al., 2009). However, more recent studies employing genetic knockout approaches in other species have demonstrated that complex social behaviors, including pair bonding and parental care, can persist in the absence of OXTR, suggesting the existence of compensatory mechanisms and alternative signaling pathways that contribute to social attachment and behavioral regulation (Berendzen et al., 2023). Importantly, these findings do not render OXTR signaling irrelevant but instead suggest that developmental compensation or redundant neuroendocrine pathways may preserve attachment-related behaviors, when OXTR is absent. However, to date the responsible mechanisms remain unidentified.

The shift from oxytocin's uniform action to its conditional action is also supported by recent empirical work demonstrating pronounced heterogeneity in responses to intranasal OXT administration, particularly in clinical populations (Audunsdottir et al., 2024), which points to the role of individual differences in treatment response. These conditional actions of OXT are similarly supported by the identification of distinct brain functional subtypes associated with differential OXT efficacy, which indicates that treatment response is linked to pre-existing network configurations rather than diagnosis alone (Liu et al., 2025). In autism, unsupervised clustering approaches have also identified subgroups that differ in their responsiveness to chronic OXT administration, reinforcing that variability in study outcome may reflect underlying biological stratification rather than inconsistent drug effects (Zhao et al., 2024).

Baseline physiological and psychological states further modulate responsiveness, with factors such as anxiety levels influencing the direction and magnitude of OXT effects (Labuschagne et al., 2010; Quintana et al., 2021). Sex-dependent differences in OXT responses add an additional layer of complexity, likely reflecting interactions between receptor signaling and sex hormone levels (Gao et al., 2016; Lieberz et al., 2020; Quintana et al., 2024). Both, OXTR gene expression in rats (Jankowski et al., 2000) and OXTR binding in prairie voles (Witt et al., 1991) have been reported to be regulated by estrogen. In women, peripheral oxytocin levels fluctuate in line with changes in estradiol levels across the menstrual cycle (Engel et al., 2019), although the role of menstrual cycle stage on the response to OXT administration in humans is currently unclear. Age also appears to play a role in oxytocin signaling, with expression level of OXTRs fluctuating over the human lifespan with the highest levels during early childhood (Rokicki et al., 2022), particularly in males. To date, very little research has directly compared the effects of OXT administration in older and younger populations (Audunsdottir and Quintana, 2022). However, preliminary research has demonstrated varied effects of age and OXT administration on connectivity, with OXT decreasing thalamus connectivity in younger adults, while increasing caudate connectivity in older adults (Xiao et al., 2024). Previous studies also reported that OXT administration improves emotion recognition in older male adults, but not older females or younger adults (male or female; Campbell et al., 2014).

Collectively, these studies support a model in which OXT effects in humans emerge from the interaction of receptor-level signaling properties, individual biological variation, and environmental context, underscoring the need for mechanistically informed and stratified approaches in both basic and translational research. These findings also suggest that human OXT effects are shaped by the same mechanistic principles that govern OXTR signaling at the cellular level. Differences in receptor structure, intracellular signaling state, and environmental context collectively determine the functional outcome. However, current experimental approaches in humans remain largely indirect, relying on systemic ligand administration without direct control over receptor-level signaling mechanisms.

## From clinical variability to preclinical experimental control: the case for receptor-level manipulation

The convergence of mechanistic and human data highlights a fundamental limitation in the current study of OXT signaling. While ligand (=oxytocin)-based approaches provide valuable insights into receptor function, they do not allow precise control over the intracellular signaling pathways engaged by the receptor. As a result, variability in receptor activation, signaling bias, and downstream pathway engagement remains largely uncontrolled. However, pharmacological approaches remain essential and can achieve pathway preference through biased, conformation-selective, or bivalent ligands, including compounds that stabilize distinct OXTR-dimer conformations and differentially recruit G proteins and β-arrestins (Busnelli et al., 2012, 2026). Importantly, the pathway bias established in controlled assays may not translate directly to *in vivo*, because receptor occupancy, exposure kinetics, tissue distribution, and cross-reactivity with vasopressin receptors remain variable. Transcript-level engineering (Figure 2) is therefore presented as a complementary strategy for testing receptor structure–function relationships rather than as a replacement for pathway-selective pharmacology.

**Figure 2:**
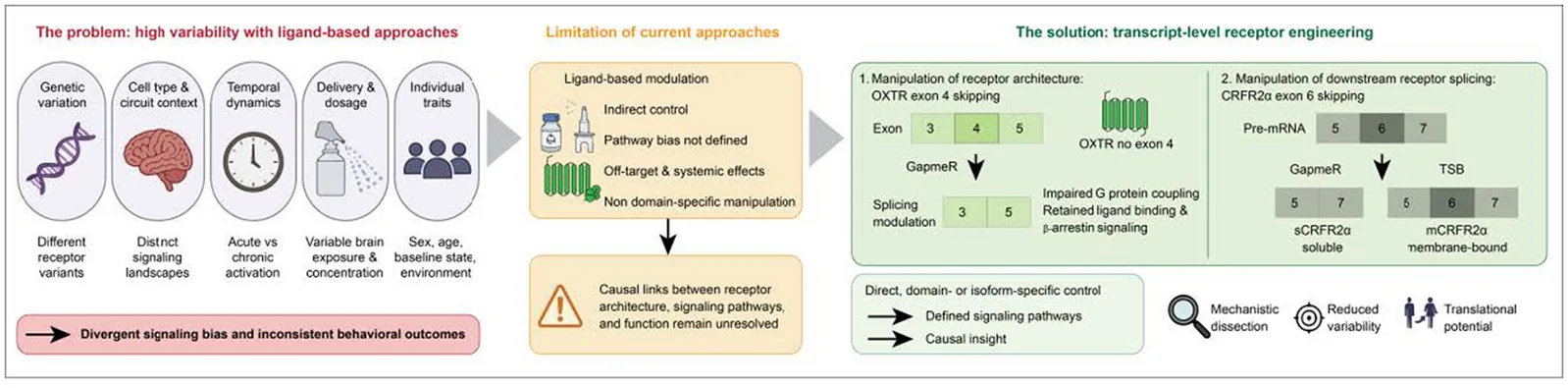
From variability to control: transcript-level receptor engineering complements current research. Ligand-based approaches are limited by genetic variation, cell type and circuit context, temporal dynamics, delivery and dosing, and individual traits resulting in signaling bias and inconsistent behavioral outcomes. Current approaches provide only indirect and non-specific control over receptor activity leaving causal links between receptor architecture, downstream signaling, and function unresolved. Transcript-level receptor engineering provides a complementing solution, enabling precise manipulation of receptor structure or isoform expression using GapmeRs. This strategy allows domain-specific control of signaling and facilitates mechanistic dissection of signaling pathways and enhances translational potential.

## Transcript-level receptor engineering

Within this context, transcript-level manipulation of receptor systems represents a critical methodological advance for dissecting OXT signaling (see Figure 3). GapmeR-mediated exon skipping enables direct modification of receptor architecture by targeting specific coding regions at the pre-mRNA level, thereby altering signaling competence in a sequence-specific manner. In detail, Jurek et al. (2022) used human MLS-402 myxoid-liposarcoma cells that overexpressed an N- and C-terminally labeled human OXTR and were cultured as 3D spheroids. GapmeR treatment induced skipping of OXTR exon 4, which encodes most of the intracellular G-protein-binding domain, thereby generating a truncated OXTR transcript and receptor. In a kinase-profiler assay, OXTR truncation was associated with differential activation of Akt, ERK1/2–RSK1/2, HSP27, STAT1/5, and CREB signaling. Spheroid organization by tight-junction formation and cellular migration from the spheroid were affected as well. In the case of the *OXTR*, removal of exon 4 generates a receptor variant that is selectively impaired in canonical G protein signaling while retaining ligand-binding capability (Jurek et al., 2022).

**Figure 3:**
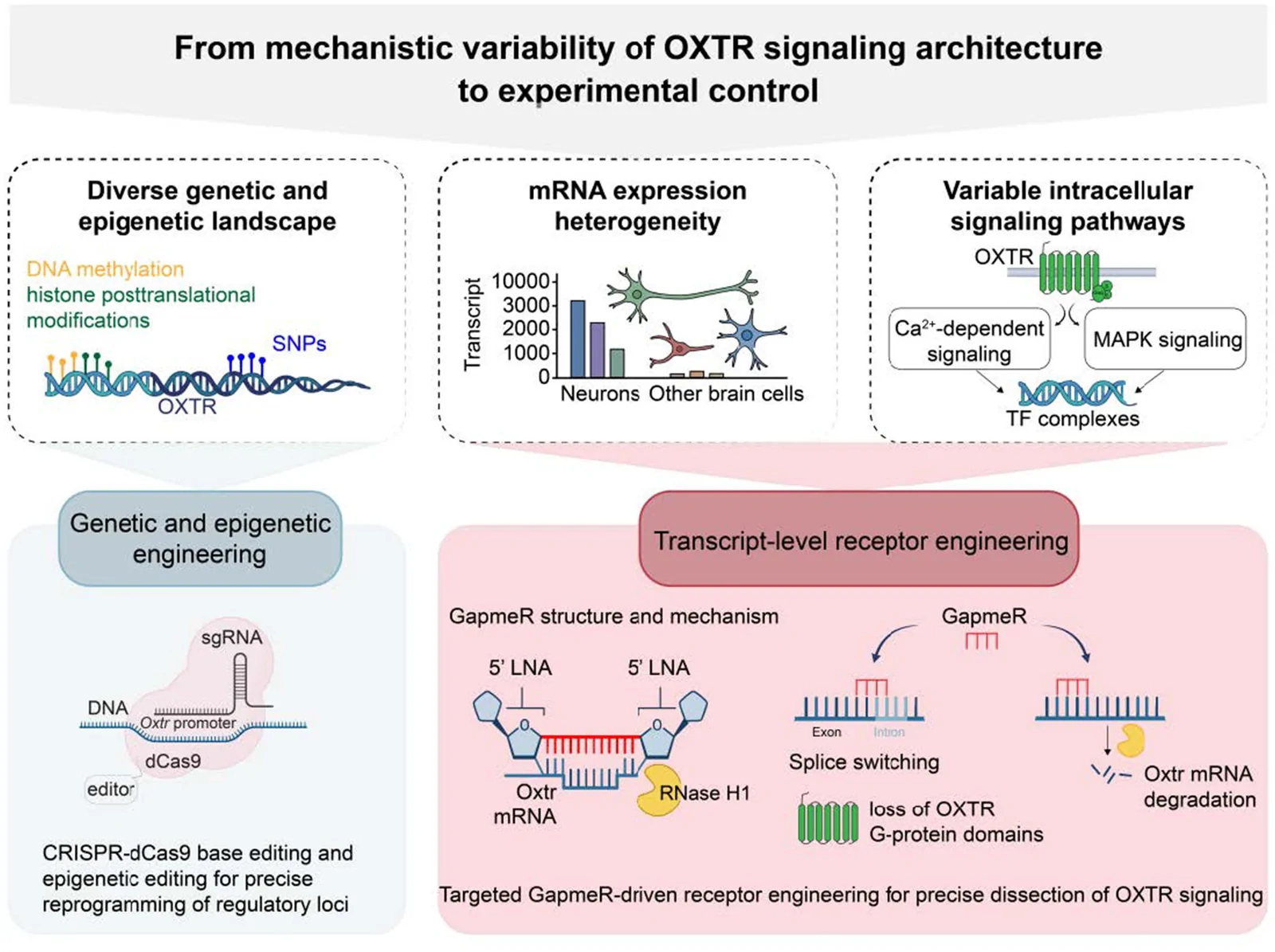
*OXTR* manipulation. This schematic highlights the need for specific genetic, epigenetic and transcript-level manipulation techniques to investigate various layers of *OXTR* regulation and signaling mechanisms when considering the OXTR system as a druggable target in psychiatric disorders. Changes in epigenetic landscape and membrane availability can be studied using locus-specific CRISPR-based epigenetic editing and base editing tools. Further downstream, mRNA expression, ligand binding, and intracellular environment are accessible using transcript-level engineering using GapmeRs (see Box 1).

Box 1 **GapmeRs at a glance.**

- Can induce RNase H1-mediated **selective degradation** of target transcripts (*OXTR)*.
- Can also be designed to alter transcript architecture through splice modulation (e.g., **exon skipping**), generating receptor variants with distinct structural and signaling properties.
- Provide a **specific** approach to investigate the relationship between receptor structure, intracellular signaling, and cellular function.
- Advantages: high sequence specificity, flexible design, **reversible action**, and the ability to manipulate both gene expression and transcript structure.
- Limitation: efficient and tissue-specific delivery remains a major challenge for *in vivo* applications.

This strategy fundamentally differs from ligand-based modulation. Rather than influencing receptor activity through extracellular binding events, transient manipulation of OXTR transcription via GapmeRs by exon skipping directly reshapes the structural determinants of intracellular signaling. As a result, it enables causal investigation of how defined receptor domains contribute to pathway selection and downstream functional outcomes. These findings support the use of exon 4 skipping to perturb receptor architecture and signaling in this engineered cellular system (Jurek et al., 2022). They do not, however, demonstrate that the truncated transcript represents a naturally occurring OXTR variant or that the same signaling profile occurs in native neurons or *in vivo*.

The broader applicability of transcript-level manipulation is further illustrated by its use in modulating receptor systems downstream of OXTR signaling. In our study (Winter et al., 2023), antisense Oligo (GapmeR-)-based approaches were employed to selectively manipulate the splicing of Crfr2α transcript in the PVN. Specifically, GapmeRs were designed to target the exon junction unique to the soluble splice variant of the CRFR2α, enabling selective reduction of sCRFR2α, while target site blockers (TSBs, designed to interfere with endogenous splice-site recognition and block the spliceosome from binding its target) were used to bias splicing toward this isoform. These complementary strategies allowed bidirectional control of receptor isoform abundance *in vivo* and provided a means to directly assess the functional relevance of alternative splicing within a preclinical stress-related receptor system.

Epigenetic editing has emerged as a powerful approach to causally link chromatin states to behavior by locus-specific manipulation of gene regulation *in vivo*. Targeted tools like dCas9-fused chromatin modifiers allow the precise modulation of promoters and enhancer regions that regulate expression of key neuronal genes, including immediate early genes and neuropeptides, leading to measurable changes in behavior (Carpenter et al., 2020; Heller et al., 2016; Rodríguez-Acevedo et al., 2026; Winter et al., 2025). Importantly, this approach is highly target-specific and reversible for controlled investigation of gene regulation without altering the underlying DNA sequence. By dissecting the functional contribution of regulatory elements, epigenetic editing provides a mechanistic framework to link transcriptional programs, including OXTR-induced signaling, to complex behaviors.

From a methodological perspective, these experiments demonstrate that splice-switching approaches using GapmeRs can be used to transiently and safely manipulate receptor systems in living, conscious rats with high specificity at the level of RNA processing. However, further validation of off-target effects, tissue exposure, toxicity, and efficacy longer than 14 days is therefore required before translational application can be considered.

## Clinical applications and limitations

Importantly, before clinical application is advised, this level of intervention requires a prior demonstration of safety, brain-wide distribution patterns and target-specificity in humans. Clinical experience with RNase H1-active antisense oligonucleotides (ASOs) shows that ASOs in general can be administered to humans but also illustrates why target-specific validation is essential. In Huntington's disease, intrathecal administration of the ASO Tominersen produced dose-dependent lowering of mutant huntingtin in cerebrospinal fluid in a phase 1–2a trial (Tabrizi et al., 2019). Inotersen, an ASO used to treat nerve damage in adults with hereditary transthyretin-mediated amyloidosis, improved neurological outcomes and quality of life (Benson et al., 2018). The ASO Tofersen reduced superoxide dismutase 1 (SOD1) in cerebrospinal fluid and plasma neurofilament light in SOD1-associated amyotrophic lateral sclerosis (Miller et al., 2022). Volanesorsen, a second-generation2′-O-methoxyethyl ASO, designed to reduce triglycerides in the blood by blocking apolipoprotein C-III production, is approved in the European Union for adults with genetically confirmed familial chylomicronemia syndrome at high risk for pancreatitis (Witztum et al., 2019). Furthermore, epigenetic editing has entered the phase of clinical trials both for small molecules targeting epigenetic enzymes (epi-drugs) as well as the first mRNA-based epigenetic editing tool (reviewed in Heller et al., 2026).

These examples establish clinical feasibility for selected RNase H1-active antisense drugs; they do not validate OXTR exon engineering, cell-type-specific brain delivery, or manipulation of human behavior.

Taken together, these approaches define a generalizable framework for transcript-level receptor engineering. GapmeR-based strategies can be applied either to directly alter the structural features of a receptor, as demonstrated for the OXTR, or to modulate the composition of receptor systems through controlled splicing. In both cases, the intervention operates upstream of protein function and allows precise manipulation of signaling cascades without relying on ligand specificity.

This level of control is particularly relevant in the context of neuropeptide signaling, where receptor systems are often closely related (e.g., OXTR and V1a/V1b) and difficult to target selectively using pharmacological agents. By enabling sequence-defined manipulation of receptor transcripts, GapmeRs provide a complementary toolset for dissecting signaling pathways and establishing causal links between receptor architecture, intracellular signaling, and functional outcomes.

## Conceptual synthesis

The integration of signaling, systems-level variability, and methodological innovation leads to a unified model of OXTR function. The receptor operates as a context-sensitive signaling hub in which structural features, activation dynamics, and intracellular pathways interact to produce diverse functional outcomes. Human variability reflects this underlying complexity, while current experimental approaches lack the precision required to fully dissect it.

Transcript-level receptor engineering, exemplified by GapmeR-induced exon skipping, and epigenetic editing, provides a means of bridging this gap. By enabling direct manipulation of receptor structure and signaling competence, it offers a powerful tool for dissecting the mechanisms underlying OXT signaling and for translating mechanistic insights into experimentally controlled systems.
